# Draft Genome Sequences of Four *Salmonella enterica* subsp. *enterica* Serovar Enteritidis Strains Implicated in Infections of Avian and Human Hosts

**DOI:** 10.1128/genomeA.01550-17

**Published:** 2018-01-25

**Authors:** Ran An, Pengpeng Lin, Salim Bougouffa, Magbubah Essack, David Boxrud, Vladimir B. Bajic, Sinisa Vidovic

**Affiliations:** a Department of Veterinary and Biomedical Sciences, University of Minnesota, Saint Paul, Minnesota, USA; b Department of Mathematics, Statistics, and Computer Sciences, University of Wisconsin—Stout, Menomonie, Wisconsin, USA; c King Abdullah University of Science and Technology (KAUST), Computational Biosciences Research Center, Thuwal, Kingdom of Saudi Arabia; d Public Health Laboratory, Minnesota Department of Health, Saint Paul, Minnesota, USA

## Abstract

*Salmonella enterica* subsp. *enterica* serovar Enteritidis is a wide-host-range pathogen. Occasionally, it is involved in invasive infections, leading to a high mortality rate. Here, we present the draft genome sequences of four *S*. Enteritidis strains obtained from human and avian hosts that had been involved in bacteremia, gastroenteritis, and primary infections.

## GENOME ANNOUNCEMENT

*Salmonella enterica* subsp. *enterica* serovar Enteritidis (here referred to as *Salmonella* Enteritidis) is a Gram-negative rod-shaped non-spore-forming bacterium capable of infecting a wide range of mammalian (1), bird (2), and reptile (3) species.

The *S*. Enteritidis strains 15-00488 and 16-03217, implicated in the infection of avian hosts, were obtained from Alabama and Wisconsin in 2015 and 2016, respectively. Two human *S*. Enteritidis strains, 2016010175 and 2011007870, were obtained from Minnesota in 2015 and 2011, respectively. The strain 2016010175 was isolated from a stool sample of a patient who suffered from gastrointestinal infection, and the 2011007870 strain was obtained from a patient who experienced bacteremia. The primary goal of this research project was to identify differences on the genome level between avian-associated (15-00488 and 16-03217) and human-associated (2016010175 and 2011007870) *S*. Enteritidis strains.

Genomic DNA was extracted using the Gentra Puregene kit (Qiagen, Inc., Valencia, CA). The whole-genome sequencing libraries were prepared using the Nextera XT DNA library preparation kit (Illumina, San Diego, CA, USA). The genomes were sequenced using an Illumina HiSeq 2500 platform with V4 125-bp paired-end sequencing chemistry. Sequencing reads were *de novo* assembled by the A5 pipeline version 5.0 (4). The draft genomes were annotated using the NCBI Prokaryotic Genome Automated Annotation Pipeline (https://www.ncbi.nlm.nih.gov/genome/annotation_prok/). PlasmidFinder 1.3 (5) and Mauve (6) were used for plasmid and single nucleotide polymorphism (SNP) identification, respectively.

The draft genomes of the avian strains, 15-00488 and 16-03217, were 4,699,188 bp and 4,771,079 bp, with average genome coverages of 60× and 183×, respectively. The genomes of the 15-00488 and 16-03217 strains resulted in 4,736 and 4,851 protein-coding sequences, respectively. The two draft genomes of the human strains, 2016010175 and 2011007870, resulted in 4,771,079 bp and 4,698,250 bp, with average genome coverages of 75× and 146×, respectively. The 2016010175 and 2011007870 strains contained 4,737 and 4,740 protein-coding regions, respectively.

Comparative genome analysis revealed that the *S*. Enteritidis strains of avian origin possessed all four genes, *galK*, *galM*, *galT*, and *galE*, that encode the enzymes galactokinase, galactose-1-epimerase, galactose-1-phosphate uridylyltransferase, and UDP-galactose 4-epimerase, respectively, which are necessary for conversion of galactose into glucose. Besides the enzymes of the Leloir pathway, the avian strains possessed the protein-coding sequences involved in acquisitions of molybdate (*modFBAE*) and iron (*fepCD*), as well as multidrug efflux (*ybhT*), and a protein of unknown function (DUF2167 domain-containing protein). Recently, it has been shown that molybdate uptake is important for anaerobic growth and multihost virulence in *Pseudomonas aeruginosa* (7, 8), which may indicate that the human-associated *S*. Enteritidis strains undergo a host restriction process compared to that of the avian strains.

In addition to loss of the genes involved in conversion of galactose into glucose and acquisition of molybdate, the human-associated strains showed a unique host-associated amino acid substitution at position 111, threonine (T) to isoleucine (I), in the gene *lpfA*, which encodes an adhesin “long polar fimbria protein A” that mediates bacterial attachment to the Peyer’s patches (9).

### Accession number(s).

The complete genome sequences of avian-associated (15-00488 and 16-03217) and human-associated (2016010175 and 2011007870) strains have been deposited in the NCBI GenBank database under the accession numbers NQNX00000000, NQUZ00000000, NQVA00000000, and NQVB00000000, respectively.
